# Analysis of the cost-efficiency of the vascular impulse technology (VIT) in the perioperative management of complex ankle fractures: results of a prospective randomised controlled trial

**DOI:** 10.1186/s13018-023-03587-x

**Published:** 2023-03-02

**Authors:** Jan S. El Barbari, Marc Schnetzke, Benedict Swartman, Paul A. Grützner, Jochen Franke

**Affiliations:** 1 Department for Trauma and Orthopaedic Surgery, BG Klinik Ludwigshafen, Ludwig-Guttmann-Str. 13, 67071 Ludwigshafen, Germany; 2 German Joint Center, ATOS Clinic Heidelberg, Bismarckstraße 9-15, 69115 Heidelberg, Germany; 3 Medical Faculty, University of Heidelberg, Heidelberg, Germany

**Keywords:** Intermittent pneumatic compression, Ankle fracture, Cost efficiency, Vascular impulse technology, Posttraumatic edema

## Abstract

**Introduction:**

Posttraumatic swelling causes a delay in surgery, a prolonged hospital stay and a higher risk of complications. Thus, soft tissue conditioning following complex ankle fractures is of central importance in their perioperative management. Since the clinical benefit of VIT usage on the clinical course has been shown, it should now be investigated whether it is also cost-efficient in doing so.

**Materials and methods:**

Included are published clinical results of the prospective, randomised, controlled, monocentric VIT study that have proven the therapeutic benefit in complex ankle fractures. Participants were allocated in a 1:1 ratio into the intervention group (VIT) and the control group (elevation). In this study, the required economic parameters of these clinical cases were collected on the data of the financial accounting and an estimation of annual cases had been performed to extrapolate the cost-efficiency of this therapy. The primary endpoint was the mean savings (in €).

**Results:**

Thirty-nine cases were studied in the period from 2016 to 2018. There was no difference in the generated revenue. However, due to lower incurred costs in the intervention group, there were potential savings of about €2000 (*p*_ITT_ = 0.073) to 3000 (*p*_AT_ = 0.008) per patient compared to the control group with therapy costs decreasing as the number of patients treated increases from €1400 in one case to below €200 per patient in 10 cases. There were 20% more revision surgeries in the control group or 50 min more OR time, respectively, and an increased attendance by staff and medical personnel of more than 7 h.

**Conclusions:**

VIT therapy has been shown to be a beneficial therapeutic modality, but it is so not only in regard to soft-tissue conditioning but also cost efficiency.

## Introduction

Ankle fractures place an enormous socio-economic burden due to their high incidence and common complications, especially in cases with complex fractures [1–5]. The sole resulting incapacity to work, which according to data from a German health insurance, leads to 1 million days of work incapacity per 100,000 insured persons, resulting in an average loss of approximately €200 per day [6, 7]. In addition, however, these fractures also place a high burden on trauma departments due to the long course to soft tissue conditioning and eventual discharge [8–10]. Developed for thromboprophylaxis in the 1980s but increasingly used for soft-tissue conditioning are systems for intermittent pneumatic compression (IPC) as is the so-called Vascular Impulse Technology (VIT). By exerting pressure on the venous plexus of the foot it is thought to result in an improved microcirculation with decongestant and antithrombotic effects, decreasing hypoxia and pain and increasing venous return [11–14]. Numerous studies—the most recent by Schnetzke et al. [15]—have already proven their clinical benefits, for the thromboprophylactic effect as well as improved decongestion, shorter hospital stays and fewer revision surgeries [15–17]. In a previous study it could be shown that administration of the VIT therapy led to a significant decreased delay to surgery of 2–3 days, a significant lower pain intensity, a significantly faster oedema reduction and a significantly lower revision surgery rate [15].

What has not been investigated, though, is to what extend these clinical advantages provide an economic effect.

In 2012, Feldman et al. wrote in their systematic review on IPC that an assessment of the economic benefit of these was urgently needed given the high initial cost of the devices compared to other modalities [18]. The only figures they found were based on a single case study of lymphedema [19]. Though the work of Thordarson already refers to the cost factor in 1997, it only mentions generally that a reduction in costs of USD 2000 could be achieved per day of shorter length of stay [20]. This thesis, however, was not based on actual study data, but on the author’s opinion. More accurate yet vague data were provided by Stöckle et al. when they compared VIT therapy with various methods of cooling in 1997 [21]. Here, they spoke of either initial costs of about $4000 per device or weekly rental costs of about $170 per device and $35 per footpad. However, an actual analysis of the total costs incurred and a comparison of these with the revenues from the different groups was not made either.

Thus, this is the first study to address this question specifically, including prospectively randomised case data and a detailed analysis of revenues and costs.

The aim of this study was to investigate whether VIT therapy leads to an economic benefit due to a reduction in costs incurred, to quantify this benefit and to extrapolate it to the economic potential outside of limiting study conditions.

## Materials and methods

In this study, the available data of 39 ankle fractures from the prospective randomised controlled trial performed at the BG Trauma Center Ludwigshafen during the period from 2016 to 2019 were analysed. The VIT study had been registered at the DRKS (german clinical trial registry, an approved primary registry in the WHO network; DRKS00010510) and the study protocol had been published a priori [22]. Sample size calculation for the clinical trial resulted in 34 complete datasets that would be necessary to show a reduction in delay to surgery of 2 days with a power of 80% and a significance level of 5%. These were patients with bimalleolar ankle fractures that could not be definitively treated surgically on the day of admission due to soft tissue swelling and received inpatient soft-tissue conditioning. Patients aged 18 to 80 years, without an injury to the contralateral extremity, were included. Patients with open fractures, local soft tissue problems (tension blisters, necrosis, compartment syndrome), or decompensated heart failure, thrombosis, or pulmonary artery embolism were excluded. After informed consent, they were randomised in a 1:1 ratio to the intervention and control group.

Patients in the intervention group received VIT therapy for soft-tissue conditioning and were instructed to use it 24 h per day preoperatively if possible and at least 6 to 8 h per day postoperatively. VIT therapy was performed using the VADOplex device (OPED GmbH, Oberlaindern, Germany). This air compressor inflates and deflates an air bubble, placed under the sole of the foot by a foot pad, to a pressure of 130 mmHg within one second at an interval of 20 s. The control group received exclusively elevation of the extremity. No further soft tissue conditioning measures were allowed to be applied in either group. Blinding throughout the clinical study was not possible due to the obvious difference in interventions. However, the primary outcome parameter and daily treatment were conducted by independent residents and consultant surgeons not involved in the study. The detailed description of the clinical study course, its results and limitations can be found in the paper previously published in 2021 [15].

After business accounting had been completed, it was now possible to use the case numbers of these patients to calculate the revenue, the detailed costs and thus the contribution margin generated in the individual cases. In addition, the same parameters were collected for all ankle fractures treated in 2018. Revenues were provided in form of the payment received by the health insurer per case and incurred costs included staff wages (physical therapy, surgical ward, anaesthesology and OR), pharmaceutical expenses and medical and non-medical infrastructural costs.

## Statistics

As a primary outcome, it was investigated whether a statistically significant difference could be found between the VIT patients and the control group regarding the contribution margin calculated as the difference between total revenues achieved and all incurred costs in every case. This difference was compared in regard of difference in number of surgeries, total OR time and care provided by medical staff in minutes. In addition, it was examined how many ankle fractures were treated at the institution in 2018 in order to extrapolate the result to the actual annual number of cases and thus more accurately assess the economic impact. The collected data are presented using appropriate descriptive statistics (mean ± standard deviation, median with Q1 and Q3). Data were analysed, according to the intention-to-treat principle (ITT) firstly and secondly, as a sensitivity analysis, using the as-treated principle (AT), according to the therapy actually performed. Two-sided *p*-values were calculated for continuous characteristics using the t-test for unequal variances or the Mann–Whitney U-test, and for nominally scaled variables using the Chi^2^ or Fischer’s exact test. Pearson’s r was calculated to analyse correlation between allocated study group and economic impact.

Statistical analyses were performed and graphics desined using the Prism program from GraphPad Software, version 8.3.1 and statistical significance was set at *p* < 0.05.

## Results

Complete data sets from 39 patients were available for analysis. No significant differences could be found in regard of demographic parameters, which are shown in detail in Table 1. According to the ITT principle 20 patients were analysed in the VIT group and 19 in the control group. However, in the sensitivity analysis, only 17 patients in the VIT group but 22 in the control group were analysed because of inadequate compliance in the use of the device in three cases. Patient demographics were comparable in terms of age, sex, injured side, fracture classification, and previous diseases.

**Table 1:** Comparison of demographical parameters

	VIT	Control	*p*-Value
Cases			
ITT	20 (51%)	19 (49%)	–
AT	17 (44%)	22 (56%)	
AO/OTA classification			
B_ITT_	6 (30%)	7 (37%)	0.741^1^
C_ITT_	14 (70%)	12 (63%)	
B_AT_	5 (29%)	8 (36%)	0.740^1^
C_AT_	12 (71%)	14 (64%)	
Age mean (SD)	51.2 (17.1)	53.2 (15.8)	0.707^2^
Sex			
Male	11 (55%)	9 (47%)	0.752^1^
Female	9 (45%)	10 (53%)	
Academic			
Yes	3 (15%)	2 (10%)	0.815^3^
No	15 (75%)	14 (74%)	
Unknown	2 (10%)	3 (16%)	
Comorbidities	9 (45%)	12 (63%)	0.341^1^
Smoker	3 (15%)	7 (37%)	0.118^1^
Side			
Right	9 (45%)	9 (47%)	> 0.999^1^
Left	11 (55%)	10 (53%)	

Values are presented in mentions with (percentage), if not stated otherwise

*ITT* Intention-to-treat, *AT* as treated

^1^Fisher ‘s exact test

^2^Student's t test for independent samples with Welch's correction

^3^Chi-square test

No significant differences could be found analysing the total length of stay (4 days, 95% CI [− 0.8; 8.9], in favour of the VIT group; *p* = 0.101) and the total revenues (comparable in both groups, *p* = 0.921; s. Table 2). A relevant difference in incurred costs of €2200 (95% CI [850; 5200]) in favour of the VIT group could be seen, but it did not reach statistical significance (*p* = 0.140; s. Table 2). These reduced costs resulted mainly from less personnel (medical, nursing and administrative services) and general infrastructural expenses. Because of the longer inpatient stay (difference between medians of 4 days) and the higher rate of revision surgeries, there were mean additional costs in the control group of €540 on the trauma ward and €690 in the OR. The costs in the OR were caused by 20% more surgeries or an average of 50 min longer OR time, respectively. The raised costs in the ward were due to a need of 360 min more attendance by medical, nursing and administrative staff and 70 min more by personnel of the anaesthesiology department.

**Table 2:** Comparison of clinical and economical parameters

	VIT	Control	*p*-Value
*Length of stay (d)*			
ITT	16.8 (5.3)	20.8 (9.0)	0.101
AT	17.1 (4.6)	20.0 (9.1)	0.214
*Revenues (TEUR)*			
ITT	12.6 (4.4)	12.8 (7.0)	0.921
AT	13.2 (4.3)	12.2 (6.8)	0.620
*Costs (TEUR)*			
ITT	9.6 (2.7)	11.8 (5.9)	0.140
AT	9.6 (2.5)	11.6 (5.7)	0.189
*Contribution margin (TEUR)*			
ITT	2.9 (3.2)	0.9 (3.6)	0.073^2^
AT	3.6 (2.9)	0.7 (3.4)	**0.008**^2^

Significant* p*-value is displayed in bold

Values are presented in means with (standard deviation). *p*-values are calculated using Student's t test for independent samples with Welch's correction. *ITT* Intention-to-treat, *AT* as treated

^2^Significance level was set at* p* < 0.05

**Fig. 1:**
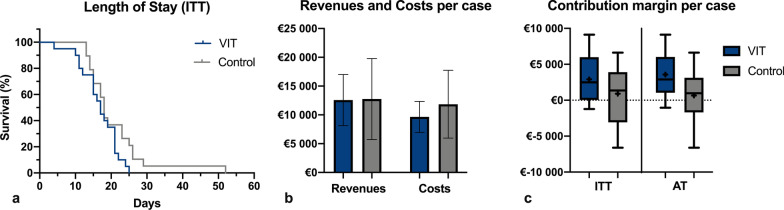
Comparison between the VIT and control group in regard of **a** the total length of inpatient stay depicted in Kaplan–Meier curves, **b** the revenues and costs per case presented in mean with standard deviation and **c** the incurred contribution margin according to ITT and AT compared by boxplots

Comparing the contribution margin showed a tendency towards a larger profit in the VIT group in the ITT-analysis with a delta of €2000 (95% CI [210; 4200], *p*_ITT_ = 0.073) and a significant difference in the sensitivity analysis with a delta of €3000 (95% CI [870; 5000], *p*_AT_ = 0.008; s. Table 2 and Fig. 1). Correlation analysis led to a Pearson’s r of − 0.42 and a *p*-value of 0.008. The post-hoc power analysis—that was applied since sample size calculation focused on the clinical outcome—lead to an achieved power of 0.8.

In 2018 a total of 354 patients who suffered an ankle fracture were treated at the institution with a mean length of stay of 13 days (5 days less than the average study patient). Both the mean costs and the revenues were also below the study average, with a difference of approximately €660 in the former and €3200 in the latter case.

In treating one patient with the VIT therapy costs of €1200 would be incurred for the intermittent pneumatic compression device, about €100 for the foot pads (2–3 pieces, depending on usage) and €75 for maintenance fees. In total, this would amount to €1400. Since the devices can be repeatedly used and the maintenance fees are calculated annually, the more patients are treated, the lesser the cost would be in the single case, reaching a steady state below €200 per case after 10 patients treated. These expenses and the extrapolation to the estimated annual cases at this institution, are detailed in Table 3.

**Table 3:** Evaluation of the acquisition and maintenance costs of VIT therapy

	Per patient	Annually^1^
Cases	1	62
Length of stay (d)	17	1054
Total period of therapy (with 6-8 h per d)	91 h	7378 h
Costs		
VADOplex device (EUR)	1200	3600
Foot pads (EUR) (2–3 per patient)	93	4728
Maintenance (EUR)	75	225
Total costs (EUR)	1368	8553
In % of contribution margin	ITT 68.4	ITT 6.9
	AT 45.6	AT 4.6

^1^Calculation based on patient data from all treated ankle fractures treated at the institution in 2018

**Fig. 2:**
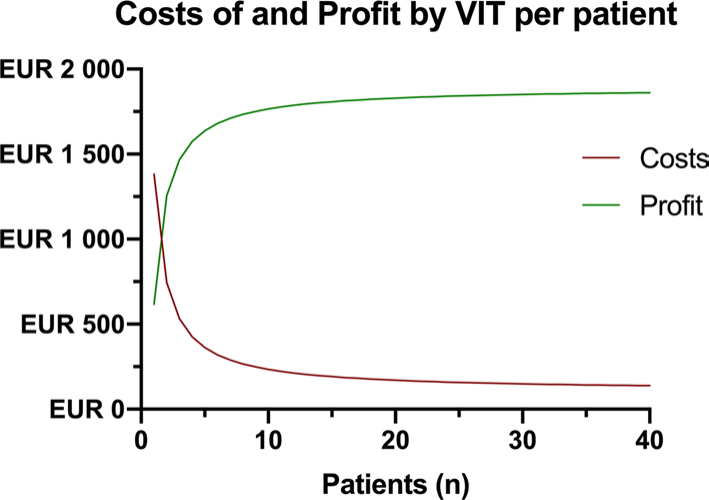
Cost-efficiency of the VIT therapy. Costs of and profit by VIT therapy are displayed per patient. As can be seen, even in the first patient a net profit of around €600 can be achieved after all therapeutic costs have been deducted, reaching a steady state of around €1800 after 10 patients

The relation between therapy costs and net profit as a function of patient numbers is displayed in Fig. 2.

## Discussion

The purpose of this study was to investigate whether treatment with an intermittent pneumatic compression (IPC) device could provide economic benefits in addition to the proven clinical benefits. The Vascular Impulse Technology (VIT) has been introduced as a method for IPC in the 1980s, primarily as a means of thrombosis prophylaxis [11, 12]. For this purpose, the devices have also been best evaluated and already represent a standard therapeutic agent in hip and knee arthroplasty [13, 14, 23, 24]. Subsequently, studies focusing on the clinical benefit have been conducted showing a reduced delay to surgery and inpatient stay, less pain and need for analgesics, and fewer complications and revisions [11–17, 25, 26]. Though some papers noted high therapeutic costs of this intervention, whether it offers an economic benefit, as could be expected from shorter hospital stays and revisions, has never been assessed. Papers addressing this topic treated it merely as a minor issue in the discussion relying on vague estimated values or solely on the author’s opinion [18, 20, 21]. An actual analysis has never been performed, this study being the first to do so.

According to these findings, a potential saving in the VIT group could be found by €2000 regarding the ITT population (*p*_ITT_ = 0.073), being statistically significant with €3000 in the sensitivity analysis, respectively (*p*_AT_ = 0.008). Upon comparison of the incurred costs of it can be concluded that with €1400 for a single patient but below €200 if more than 10 patients are treated each year, the VIT therapy resembles a cost-efficient therapeutic modality, in a single case but even more in repeated usage.

However, transferring it to the average and more benign ankle fractures is difficult and only possible to a limited extent at all. As could already be demonstrated in the shorter length of stay and the lower revenues and costs of all patients admitted with ankle fractures in general, the study patients suffered more severe fractures resulting in strained soft tissue conditions and thus being more prone to complications. Since the dislocated bimalleolar fractures, as included in the prior study, have an incidence of 15–20% among all ankle fractures it was assumed that of the 354 patients admitted in 2018 62 would have suffered from such an injury [2]. In these 62 cases a possible cost saving of €120,000 (ITT) or €185,000 (AT) could be achieved.

Another potential factor for the cost-effectiveness of VIT therapy is that this therapy reduces the median length of stay of these patients by four days (see Fig. 2). German health insurance companies pay hospitals according to diagnosis-related groups (DRG), resulting in fixed budgets depending on the injury sustained. The complexity and prolonged length of stay for complex joint fractures mean that the costs are not covered by the flat-rate payment by the health insurance funds. Resulting from this: the highest profit is made if the patient is discharged when the average length of stay (16 days for ankle fractures) is reached. As soon as the upper limit is exceeded, additional hospital days no longer lead to higher remuneration and have a negative impact on hospital accounting. In the study group, no case of exceeding the upper limit was observed—as did in the control group in 17% of their cases—and the mean length of stay corresponded to the average length of stay resulting from this DRG.

Concluding from a difference of 50 min OR time and around 7 h less personnel resources compared to incurred costs of below €200 if used frequently, the VIT therapy is a cost-efficient therapeutic modality in soft-tissue conditioning.

## Limitations

The first limitation to be mentioned is that this was not a blinded study, as the intervention using the intermittent pneumatic compression device could not be simulated in placebo form for the control group. Thus, a bias because of any influence by the treating physicians cannot be ruled out completely. What was also not taken into account was the time and resources needed to ensure adequate treatment adherence, i.e. instruction of device usage, answering questions that arose or removing and reapplying the pad if dislocated. However, compared to the actual costs incurred and estimated, this would only be a small amount, assuming a time of 20–30 min per patient.

The main limitation is of course that the transfer of the potential savings from a controlled study population upon everyday treatment is only an extrapolation. If the possible savings reach the estimated number needs to be proved in further studies. However, that there is an actual possible economic benefit had been shown by the data of the RCT that had been conducted before this analysis was even planned. So there’s only a limited chance that an observer bias for example could have influenced the data in this regard. Especially figures like OR time and number of revision surgeries as objective parameters should not be susceptible to being influenced by this.

## Conclusion

VIT therapy is not only an efficient adjunct to current methods for soft tissue conditioning and can improve patient care but is also cost-efficient, even if used infrequently.

## Data Availability

Raw data were generated at the BG Trauma Center Ludwigshafen. These and the derived data supporting the findings of this study are available from the corresponding author on request.
